# Effect of Scanning Strategy on the Microstructure and Triboperformance of FeNiCrMo Coating Manufactured by Plasma Transferred Arc

**DOI:** 10.3390/ma16175931

**Published:** 2023-08-30

**Authors:** Botao Xiao, Shang Li, Xianglin Song, Qiwen Huang, Jin Lou, Jun Fang, Pengfei Hou, Huatang Cao

**Affiliations:** 1 Jiangxi Key Laboratory of Material Surface Engineering, Jiangxi Science and Technology Normal University, Nanchang 330099, China; 2 State Key Laboratory of Materials Processing and Die & Mould Technology, School of Materials Science and Engineering, Huazhong University of Science and Technology, Wuhan 430074, China; 3 Wuhan Huacai Surface Technology Co., Ltd., Wuhan 430074, China

**Keywords:** FeNiCrMo coating, microstructure, triboperformance, plasma transferred arc, ductile iron

## Abstract

To increase the coating thickness and service life of the FeNiCrMo coating, a plasma transferred arc (PTA) double-track alloying technique was employed to enhance the surface triboperformance of the ductile iron. Optical microscopy (OM), X-ray diffraction (XRD), electron probe X-ray microanalyzer (EPMA), scanning electron microscopy (SEM), transmission electron microscopy (TEM), Vickers hardness tester, and tribological tester were subsequently used to evaluate the effect of the double alloying treatment tracks on the microstructure and triboperformance of the coating. The results indicate that the content of the cementite in the sample with a double-track treatment increases 3.90 wt.% and the content of the martensite decreases 13.04 wt.% compared with the sample with a single-track treatment, which results in the maximum microhardness of the sample fabricated by double track increasing from 837 ± 10 HV_0.2_ for the sample fabricated by single track to 871 ± 7 HV_0.2_. Thus, the wear rate is lower than that of the sample with a single-track treatment. In addition, the distribution of alloying elements is more uniform and coating thickness is higher in the double track than those of the single-track-treated one. Therefore, the double-track PTA alloying treatment is favored for hardfacing ductile iron with a FeNiCrMo alloy coating due to its enhanced triboperformance and longer service life.

## 1. Introduction

Surface engineering is the use of physical, chemical or mechanical processes to modify the surface state of the alloy matrix, chemical composition, microstructure and stress state so that the alloy surface has different properties from the matrix, such as wear resistance, corrosion resistance, oxidation resistance, etc., which can extend the service life of materials and components. At present, many effective surface modification methods have been developed. In addition to traditional surface modification technologies such as shot peening, surface quenching, chemical heat treatment, etc., high-energy beam surface modification technologies containing a laser beam [1], electron beam [2] and plasma beam [3] have been widely used in the past decades. Among them, the plasma transferred arc (PTA) has many advantages of minimal thermal deformations, metallurgical bonding and refined grains under rapid cooling. It has a great application potential, for example, for remelted coatings [4,5], clad coatings [6,7] and alloyed coatings [8,9,10,11,12]. Previous results demonstrated that PTA treatment enhances the targeted surfaces’ hardness, wear resistance and other mechanical properties. Cheng et al. [8] reported that the surface hardness of the remelted surface by PTA is 2.19 and 1.96 times greater than that of the substrates of gray and ductile iron, respectively. Pan et al. [10] and Zhao et al. [11] manufactured Fe-Cr-C and Cu cladding layers, respectively, using PTA to improve the frictional properties and the wear resistance of the alloy. In addition, alloy coatings, such as CrNiMo and CrNiMoCu [6], high vanadium high-speed tool steel [12] and other composite coatings, were fabricated on the surface of ductile cast iron and compacted graphite iron by PTA to enhance their wear resistance.

The CrNiMo is a traditional material and its use for cast structures was presented since the 1930s [13]. Since its first appearance, it was applied to a component or substrate to create a protective coating, which can be used for medium- and large-sized gears [14]. At present, numerous surface strengthening methods, such as thermal spraying [15,16], plasma spraying [17,18] and high-energy surface modification [6], have been used to fabricate NiCrMo coatings on metal parts or substrates. Walsh et al. [15] investigated the influence of the Ni-Cr-Mo alloy coating formed on mild steel and indicate that the electrochemical activity of the coating fabricated by thermal spraying was higher than that of the bulk alloy, which is attributed to the splat boundaries formed. Moreover, Fe-Cr-Ni-Si-B-C and Fe-Cr-Ni-Mo-Si-B-C coatings were formed on the surface of low carbon steel (Fe52) plates, and their effect on the abrasive resistance was studied; they suggested that the Fe-Cr-Ni-Mo-Si-B-C coatings formed by thermal spraying possess higher sliding wear resistance than that of a Fe-Cr-Ni-Si-B-C coating because its nano-hardness is higher than that of the latter [16]. Wen et al. [17] explored the abrasive resistance and corrosion resistance of the remelted NiCrMoY alloy coating fabricated by plasma spraying and reported that the abrasive resistance of the NiCrMoY coating was improved due to the formation of CrB, Cr_23_C_6_ and some other new phases after remelting. In addition, a post-spray shot peening treatment increases the density of the bulk-like NiCr-20Mo coating, which can enhance the corrosion resistance of the plasma spray NiCr-20Mo coating [18]. As mentioned above, the composition of CrNiMo is a promising coating material that has been popular with researchers since its emergence, which can be used to improve the abrasion and corrosion resistance of the alloy.

Previous results show that PTA is suitable for remelting, cladding and alloying on the surface of cast iron to improve its surface properties, but PTA has not received enough attention as a FeNiCrMo coating on the surface of ductile iron to improve its abrasive resistance. In addition, the thickness of the alloy coating produced by a single-track PTA treatment should not be sufficient as this would result in the formation of cracks and other defects, which could not significantly increase the service life of the components or substrate. Therefore, in this study, FeNiCrMo alloy coatings were prepared on ductile iron using double-track PTA treatments inspired by additive manufacturing. Then, the microstructure and triboperformance of the alloy coating fabricated by double-track treatment were studied and compared with single-track treatment to investigate the scanning strategy increasing the thickness of the alloy coating under fewer defect conditions.

## 2. Experimental Procedure

**Figure 1:**
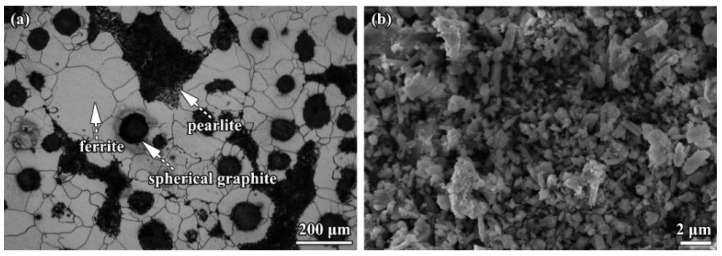
Microstructure of materials: (**a**) substrate and (**b**) alloy powder morphology of the coating after drying.

The substrate was ductile iron QT600-3 with chemical compositions of 3.74 wt.% C, 2.40 wt.% Si, 0.55 wt.% Mn, 0.017 wt.% S and 0.042 wt.% P; its microstructure, consisting of pearlite, ferrite and spherical graphite, is shown in Figure 1a [12].

For the preparation of alloy coatings, a commercially available metal powder containing nickel, chromium and molybdenum (Hebei Lebo Metal Material Technology Co., LTD, Xingtai, China) was utilized. The powder’s particle size ranged from 45 to 55 μm. Furthermore, 0.9 g of a rare earth fluoride mixture containing rare earth oxide (REO), CeO_2_ and F was mixed with 30 g of metal powder and served as the binder. The chemical composition of rare earth fluoride is shown in Table 1 and the constituent (wt.%) of the powder was Ni67Cr27Mo6. To refine and homogenize the mixture powder, the powder was ball-milled in absolute ethanol for 24 h, and the ball-to-powder ratio is 15:1 [19]. Then, the alloying coating was obtained. After taking an amount of alloy coating and drying, the powder morphology was examined by scanning electron microscopy (SEM, Leo 1530 FEG, Carl Zeiss, Jena, Germany), and is shown in Figure 1b.

It can be seen that the size of the alloy powder was refined to some degree, and the minimum size approached 500 nm. However, the alloy powders are not uniform in size, and the maximum size is about 5 μm. The surface was pre-coated with a thickness of approximately 90 μm to avoid the induced defect of the powder alloy before using PTA with the following treatment parameters: arc current 50 A, scanning speed 20 mm/s, plasma torch diameter 4 mm, working distance 4 mm and plasma gas flux (Ar) 0.033 L/s, respectively. The PTA treatment diagram is shown in the reference [20].

**Figure 2:**
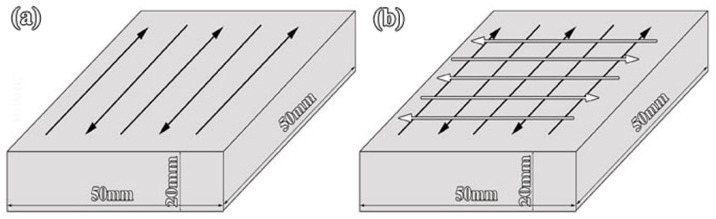
Schematic diagram of the alloying treatment approaches of PTA: (**a**) the sample fabricated by single track, (**b**) the sample fabricated by double track.

Samples were prepared following a single and double treatment track, and the PTA treatment approaches of the two samples are shown in Figure 2. Namely, a single track was obtained by a paint alloy coating and a PTA scan; double track was obtained by two paint alloy coatings and two PTA scans (the second PTA scan was perpendicular to the first plasma arc scan track); two specimens were then cut from the center of the samples using a wire-cutting machine. Then, they were ground and polished to prepare the specimens for phase analysis by X-ray diffraction (XRD) and microstructure observation by optical microscopy (OM), electron probe X-ray microanalyzer (EPMA, Shimadzu EPMA-1720, Shimadzu Co., Tokyo, Japan), energy dispersive spectroscopy (EDS, INCA EDS, Oxford Instruments Co., Ltd., Tokyo, Japan) and transmission electron microscopy (TEM, FEI Tecnai G2 F30). The XRD analysis was performed with a scanning speed of 5°/min over the range of 20–120°, and the composition of each phase was evaluated semi-quantitatively. Cross-sectional surfaces of samples were etched with a 4% nitric acid/alcohol solution [21]. The microhardness distribution across the cross-section of the treated substrate was measured using HV1000A with a load of 1.96 N, a dwell time of 15 s and an interval of 0.1 mm. The tribological tests were conducted at 20 N normal load, and a constant stroke was 10 mm at a frequency of 500 rpm using the MFT-4000 wear testing machines under dry sliding conditions at room temperature (ASTM G119-09 (2016) Standard Test Method for Wear Testing) [22]. The sliding time of each test was typically 3600 s. The disc was composed of the PTA-alloyed layer, whereas the counterpart was a Si_3_N_4_ ceramic ball with a diameter of 6.3 mm. Finally, worn surfaces were measured with a non-contact optical profilometer to observe the morphologies of the wear scar and calculate the wear rates based on Equation (1) [23], with the measured area positioned in the center of the wear scar.

K = S × L/(P × d)(1)

where K is the wear rate (mm^3^ N^−1^ m^−1^), S is the cross-sectional area at the middle of the wear scar (mm^2^), L is the wear scar length (L), P is the normal load (N) and d is the total sliding distance (m).

## 3. Results and Discussion

### 3.1. Forming Features

**Figure 3:**
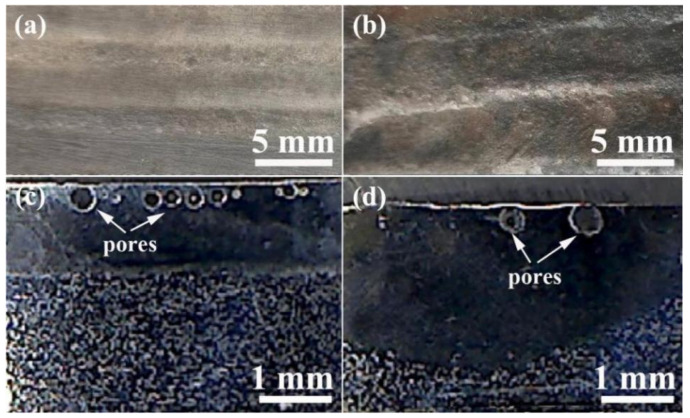
OM image of surface macroscopic morphologies and cross-section of alloy coating treated by PTA: (**a**,**c**) the sample fabricated by single track, (**b**,**d**) the sample fabricated by double track.

Figure 3 shows the OM of the surface macroscopic morphologies and a section of a PTA-treated alloy coating. As shown in Figure 3, the surface macroscopic morphologies of samples with a single- or double-track treatment are smooth and free of the defects of pores or cracks from the top surface view, which is similar to reference 14. In addition, as shown in the cross-section images of Figure 3c,d, there is gas porosity present in the subsurface. It is well known that CO or CO_2_ gas is generally produced by the reaction of spheroidal graphite and oxygen at elevated temperatures [24,25,26]. If the gas formed during the surface alloying treated by the PTA does not have sufficient time to escape from the top of the alloy coating due to the rapid solidification of the molten pool, pores could form in the alloy coating, which is a primary factor in the formation of subsurface blowholes in the sample. On the other hand, the protective argon gas during the PTA treatment is another reason for the gas formation, although it has less impact on the formation of pores. Compared to the macroscopic morphologies of the cross-sections in both samples, no evident cracks were found in the PTA-treated samples. The crack is a challenging problem to resolve in alloying coatings fabricated by welding [27] or PTA alloying [6]; neither single- nor double-track-treated samples revealed any cracks in the alloy coating. The reason might be due to the high martensite content in the alloy coating fabricated by single- or double-track-treated samples. The lattice constants of the martensite are similar to the austenite [28], which probably results in a small thermal stress between the austenite and the martensite. Although there are many references on the remelting [4,5] and alloying [6,7,8,9,10,11,12,13,14] of ductile cast iron or gray cast iron by plasma transferred arc, researchers have paid more attention to the effect of the parameter during the process of plasma transferred arc on the microstructure and properties of the remelting coating or alloy coating, and similarly this type of work may be the first time and it cannot be compared with the data of the published reference.

### 3.2. Microstructure and Phase Analysis

**Figure 4:**
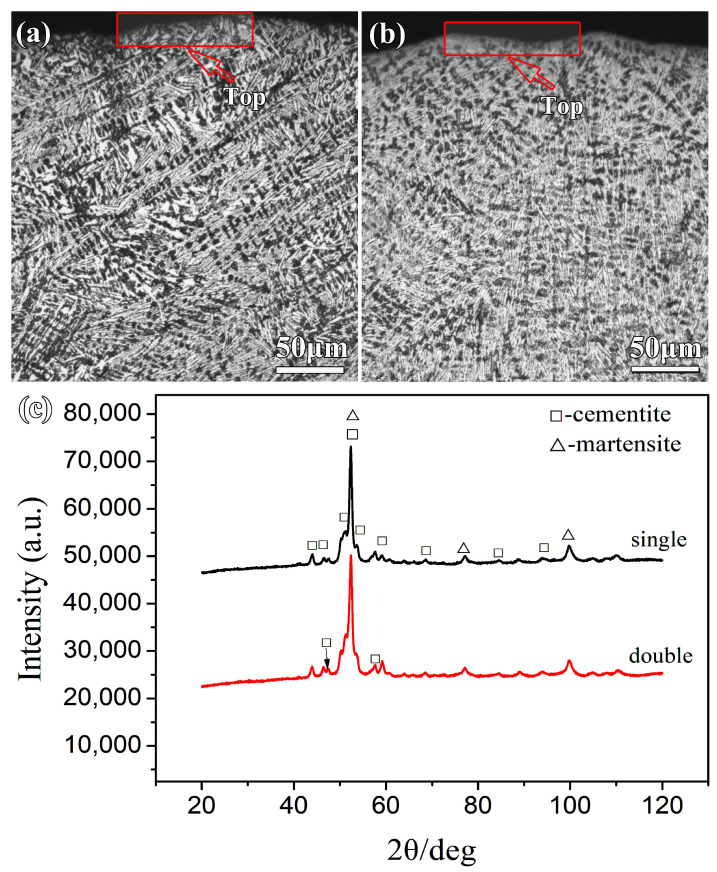
(**a**,**b**) Show the OM of the cross-sectional microstructure in samples fabricated by single and double track, (**c**) XRD patterns of the coating surface.

To analyze the effect of the treatment strategy on the microstructure of the alloy coating, the cross-sectional microstructures of FeNiCrMo coatings were observed by OM, as shown in Figure 4a,b. It can be seen that ledeburite formed in the coatings of the alloys. Samples fabricated by single track and double track show that some primary austenite was transformed into columnar dendrites containing the ledeburite. The reason is that the alloying processed by PTA induced non-equilibrium solidification and self-quenching [6], and it is well known that ledeburite is a microstructure formed during non-equilibrium solidification [29]. Compared with the sample fabricated by single track, the microstructure in the sample fabricated by double track did not change much. It can be seen that double-track treatment does not change the solidification features of the alloy coating. Besides, the sample fabricated by single track has more prominent primary dendrites than the double-track sample. In theory, this can be attributed to the solidification rate of the alloying coating and the function of alloying elements. The solidification rate of the top of the alloying coating is quick [16] and then the undercooling phenomenon presents [30], resulting in the formation of primary dendrites. According to reference [31], molybdenum added to the alloy coating inhibited the formation of primary dendrites, so the more tracks of alloying treatment there are, the higher the molybdenum content, the less developed are the primary dendrites formed.

The semi-quantitative phase analysis of the microstructure is listed in Table 2. As can be seen from Table 2, there was a large account of cementite and a small amount of martensite in the sample fabricated by double track, while there was a small amount of cementite and a large amount of martensite in the sample fabricated by single track. Carbides were typically harder than solid solutions [32], from which it can be inferred that the wear resistance of the sample fabricated by double track is higher than that of the sample fabricated by single track.

**Figure 5:**
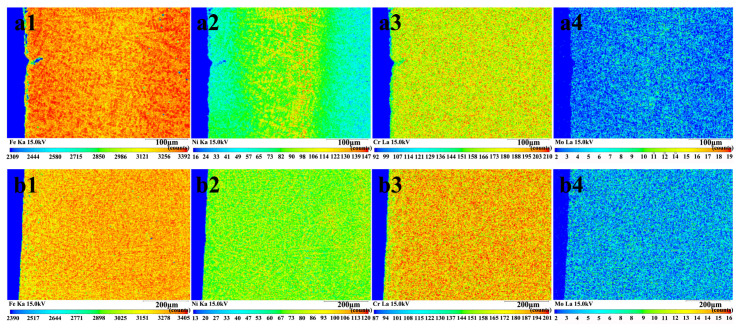
EPMA mapping of the cross-section of the alloy coating: (**a1**–**a4**) showing the elemental distribution of Fe (**a1**), Ni (**a2**), Cr (**a3**) and Mo (**a4**) in the sample fabricated by single track, and (**b1**–**b4**) showing the elemental distribution of Fe (**b1**), Ni (**b2**), Cr (**b3**) and Mo (**b4**) in the sample fabricated by double track.

Figure 5 shows the EPMA mapping of the cross-sections of samples fabricated by single track and double track to show the distribution of the elements in the cross-sections of the FeNiCrMo coating. Due to the substrate’s diluting effect, the iron, chromium and molybdenum concentrations in the coatings were low. Moreover, nickel was observed to form discrete zones in the sample fabricated by single track, while the chromium and molybdenum were evenly distributed (Figure 5a). The phenomenon of alloying element segregation has not been found in previous studies [17,18,19]. This is mainly because of the melting point difference of alloying powders [33]. In the present work, the elements with nickel, chromium and molybdenum were selected for alloying. The melting point of nickel, chromium and molybdenum is 1445 °C, 1857 °C and 2620 °C, respectively. Compared with chromium and molybdenum, nickel has the lowest melting point and tends not to form cementite. Thus, the nickel element in the alloy powder will melt first and solidify last, which readily forms segregation zones in samples fabricated by single-track alloy treatment. The microstructure characteristic of the alloy coating influences the distribution of the nickel element. During alloying, nickel is typically easily distributed in dendrites [33]. According to the phase diagram of the Fe-C alloy, the microstructure of dendrites is mainly derived from primary austenite. Therefore, nickel element segregation is promoted during the formation of the developed dendrites. In addition, there is no discernible segregation or enrichment of chromium or molybdenum in the coating, presumably because these elements were added in much smaller amounts during the coating preparation. Nonetheless, no nickel, chromium or molybdenum segregation or enrichment was observed in the sample fabricated by double track, as shown in Figure 5b. The double treatment may have altered the coating’s elemental content, solidification time and phase transition point. Additional studies are required to validate the specific causes.

**Figure 6:**
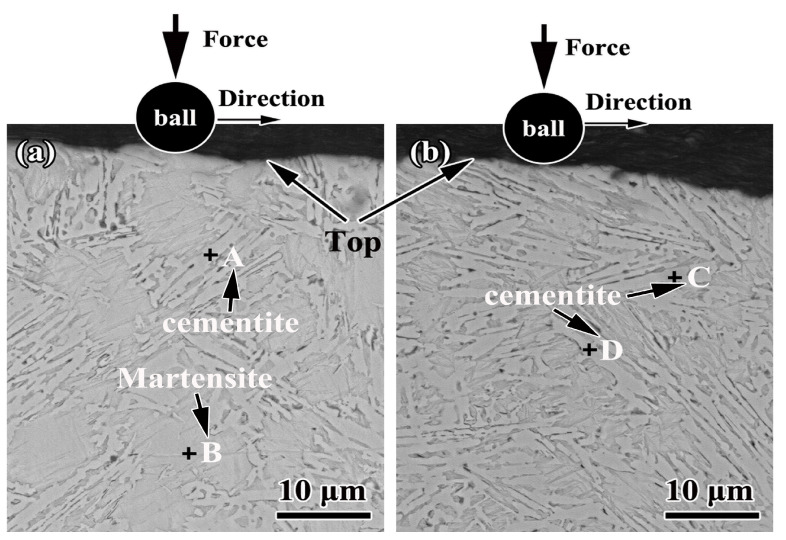
Secondary SEM images showing the microstructure of the cross-section of the alloy coating: (**a**) the sample fabricated by single track and (**b**) the sample fabricated by double track.

Figure 6 shows the SEM image of the cross-sections of the sample fabricated by single track and double track, while Table 3 displays the results of the EDS analysis at various locations in Figure 6a,b. Figure 6 illustrates that the microstructures of samples fabricated by single track and double track are identical, containing martensite formed from the transformation of the primary austenite. According to the carbon equivalent equation: CE = %C + 0.28% Si [34], the CE of the substrate is 4.54, which corresponds to hypereutectic cast iron. Due to the presence of nickel, chromium and molybdenum in the alloy coating, the CE of the alloy coating increases further, as predicted in reference [35]. According to the phase diagram of the Fe-C alloy, the carbide precipitates first from the molten pool during solidification, followed by the formation of ledeburite as the molten pool is cooled to the eutectic point. However, the actual solidification of the molten pool does not correspond to the analysis performed theoretically, i.e., the primary phase of precipitation of the molten pool is not carbide, but rather primary austenite, as shown in Figure 6. The reason is that the increase of the cooling rate influences the precipitation of the primary dendrites [6,29].

In addition, it can be seen from Table 3that the EDS of the four points A–D show that there are small amounts of alloying elements both on the cementite surface and in the ledeburite. To reduce the heat input during alloying, coatings with a thickness of 90 μm are applied during alloying, which induces the minor contents of chromium and molybdenum in the alloy coating.

**Figure 7:**
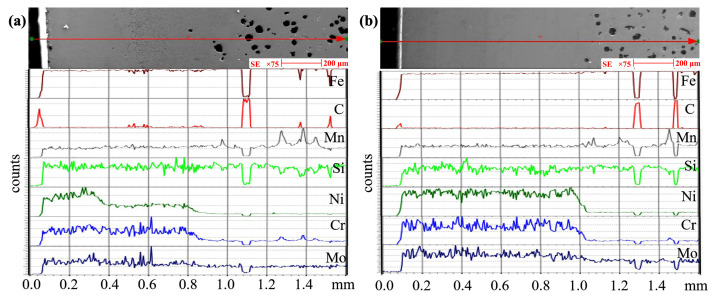
SEM micrographs and EDS line of the cross-section: (**a**) the sample fabricated by single track and (**b**) the sample fabricated by double track.

Figure 7 displays the SEM micrograph and EDS line of the cross-section in the sample to reveal the principal elements of the alloy coating. It can be seen that three areas form on the surface of ductile iron: the alloying zone (AZ), the remelted zone (RZ) and the substrate. The thinner AZ was in the sample fabricated by single track, and a higher alloying element content will result in a larger difference between the two samples. The AZ thickness of the sample fabricated by single track was approximately 720 μm, whereas the AZ thickness of the sample fabricated by double track was approximately 900 μm. Compared with the sample fabricated by single track, the AZ thickness of the sample fabricated by double track was increased by 25%. This result is also quite different from that in Figure 3c,d in that the coating thickness of the sample fabricated by double track is about twice that of the sample fabricated by single track. The reasons can be attributed to two factors: firstly, the statistical coating in Figure 3 consists of alloy coating and remelting coating. The focus here is only on the thickness of the alloy coating; secondly, it may be caused by the grinding process used to reduce the surface roughness before SEM observations, where the surfaces of samples fabricated by single and double track were ground to remove the subsurface blowholes. The precision of the grinding process is the main factor that causes the thickness of the alloy coating of the sample fabricated by double track to be only 1.25 times that of the sample fabricated by single track. The increase in alloy coating thickness was conducive to prolonging the wear resistance of service parts. In addition, the nickel element segregation in the sample fabricated by single track is consistent with the EPMA mapping results, whereas the carbon content in the sample fabricated by single track exhibits compositional fluctuations, as shown in Figure 7a. This may be because nickel is an element that promotes graphitization [36].

**Figure 8:**
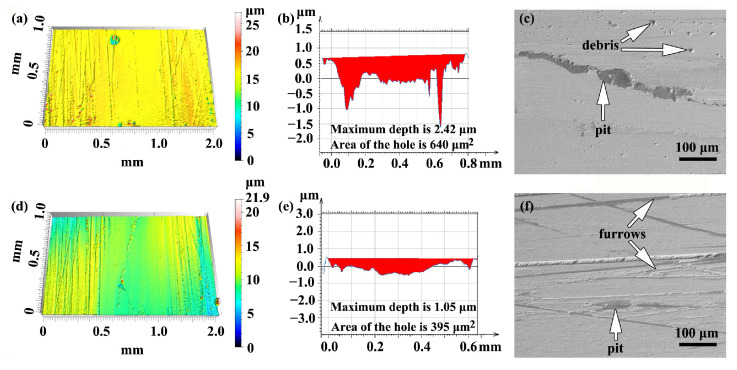
The 3D morphologies of the dry wear tracks: (**a**–**c**) the sample fabricated by single track, and (**d**–**f**) the sample fabricated by double track.

Figure 8 shows the three-dimensional morphologies and surface wear patterns of samples fabricated by single and double track. As shown in Figure 8, the dry tribological test of the sample fabricated by single track produced a deep pit with a maximum depth of 2.42 μm and a large hole area of 640 μm^2^, whereas the sample fabricated by double track produced a shallow pit with a maximum depth of 1.05 μm and a small hole area of 395 μm^2^. The difference between the peak and valley in the sectional view of the sample fabricated by double track was less than that of the sample fabricated by single track. Therefore, it can be demonstrated further that the sample fabricated by double track exhibited higher wear resistance than the sample fabricated by single track. In addition, the cross-section of the sample fabricated by single track was smooth, which points to both abrasive and adhesive wear formed during the dry tribological tests [10], as shown in Figure 8c. The furrow cross-section of the sample fabricated by double track was rough, suggesting that abrasive wear was the predominant wear mechanism [37], as shown in Figure 8f. Compared with reference [38], it can be seen that the smooth width and depth of the grooves on the surface of the FeCrNiMo coating treated by PTA were lower than those of TiC particle reinforced ductile iron, no matter whether formed by single- or double-track treatment, which presented a better abrasive resistance than that of reference [38]. It can be further proved that the wear resistance of the ductile iron was enhanced using the prepared FeCrNiMo coating on it.

**Figure 9:**
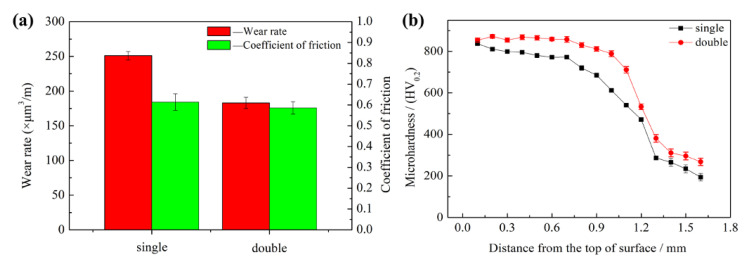
The properties of the samples fabricated by single and double track: (**a**) wear rate and mean coefficient friction; (**b**) microhardness across the cross-section.

The wear rate of the alloy coatings was calculated based on Figure 8 and Equation (1), and is shown in Figure 9a. As can be seen, the sample fabricated by double track had a lower wear rate and coefficient of friction (CoF) than the sample fabricated by single track, although the difference in CoF between the two samples was not significantly apparent. As shown in Figure 9b, the maximum microhardness of the samples fabricated by single track and double track was 837 ± 10 HV_0.2_ and 871 ± 7 HV_0.2_, respectively, and the maximum microhardness of the sample fabricated by the double track increased by 4.06% compared to that of the single track. The reason is that the cementite content in the samples fabricated by double track is larger than that of the samples fabricated by single track. Compared with reference [6], the maximum microhardness of the samples fabricated by single track and double track is higher by about 4.63% and 8.88%, respectively. The cause is that the CE of the ductile iron used in this paper is higher than that in reference [6], resulting in more cementite content in the alloy coating. Besides, it can be seen from the microhardness curve in reference [6] that the thickness of the alloy coating fabricated by double track is significantly greater, with an increase of about 66.7%. Thus, the use of the double-track PTA treatment to fabricate the FeNiCrMo coating is an effective scanning strategy to increase the thickness of the alloy coating.

**Figure 10:**
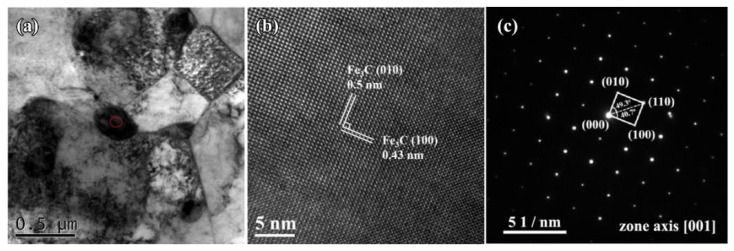
TEM micrographs of the cross-section of the sample fabricated by double track: (**a**) bright-field TEM images, (**b**) the HRTEM image along the zone axis of [001], (**c**) the selected electron diffraction pattern (SAED) in red circle of (**a**).

**Figure 11:**
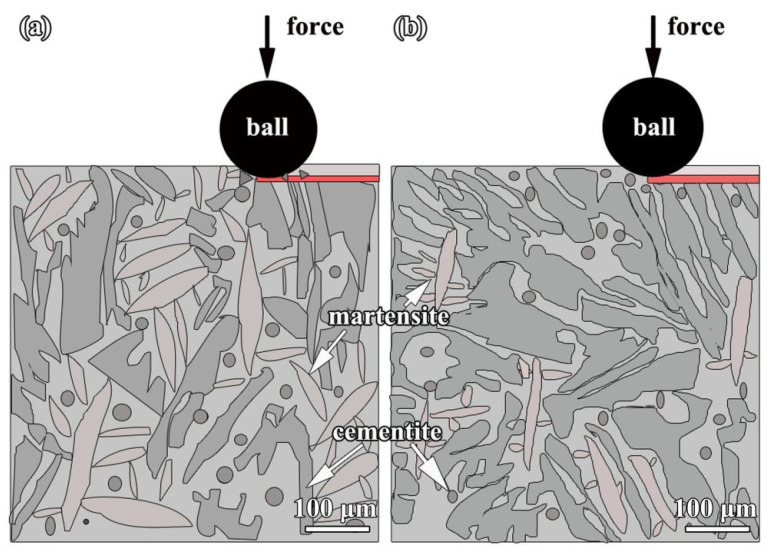
Schematic diagram of coating wear-resistant mechanisms: (**a**) the sample fabricated by single track and (**b**) the sample fabricated by double track.

Figure 10 shows TEM micrographs of the sample fabricated by double track. In Figure 10a, the grain size ranged from approximately 500 nm to 1 µm, indicating that the crystallization of the samples was satisfactory. In addition, the region denoted by the red circle in the image may be the precipitated phase in the alloy coating, which was between 400 and 500 nm in size and located at the grain boundaries. In addition, Figure 10b shows a high-resolution transmission electron micrograph (HRTEM) of the sample, which reveals multiple lattice fringes with variable spacings, which correspond to cementite of the orthorhombic system (PDF# 03-0411). Cementite is usually lamellar like and granular like [39], but it is found in this paper that cementite is a granular belonging to the secondary carbide, rather than lamellar -like, which is similar to reference [40]. In addition, granular cementite precipitated along lath grain boundaries, which has been observed in reference [41]. Besides, the lattice fringes with a spacing of 0.43 nm and 0.5 nm could correspond to the (100) and (010) crystal planes of cementite, and the angle between the (100) and (010) crystal planes was 90°. Figure 10c displays the selected electron diffraction (SAED) pattern of the cementite. It demonstrates that the characteristics of the diffraction point present a feature of a single crystal. The adjacent diffraction spots corresponded to the crystal planes (100) and (010) of cementite (PDF#03-0411). Simultaneously, the diffraction spots corresponding to the (110) crystal plane can be determined, and the angles between the (110) crystal plane and the (100) and (010) crystal planes were 40.7° and 49.3°, respectively. Simultaneously, the crystal zone axis orientation of the tested sample was [001]. According to the previous results and analysis, the wear rate of the sample fabricated by double track is lower than that of the sample fabricated by single track. Consequently, the wear resistance of the sample fabricated by double track is higher than that of the sample fabricated by single track, and the wear-resistant mechanism is shown in Figure 11. The enhancement of the wear resistance of the sample fabricated by double track can be attributed to the content of cementite being larger than that of the sample fabricated by single track. In the meantime, a self-quenching occurs [7,8] during the alloying fabricated by PTA, and the microstructure includes cementite and martensite. Otherwise, the content and distribution of cementite are other factors affecting the hardness and wear resistance of the alloy coating.

Combined with Figure 6 and Figure 10, the microstructures of both coatings include cementite and martensite [38,39,40], and the cementite precipitated was granulose. In the sample fabricated by single track, the cementite precipitated along lath grain boundaries and a large amount of martensite was transformed and surrounded by the cementite. However, the cementite content on top of the sample fabricated by double track was increased due to the increase of the CE. It is well known that both martensites defined a supersaturated solid solution of carbon atoms in α-Fe and the cementite functions as a strengthening phase to achieve solid solution strengthening and dispersion strengthening, which favorably enhance the hardness and wear resistance of the alloy coating. Compared to the sample fabricated by single track, the content of the block-like cementite in the sample fabricated by double track is larger and the amount of austenite transformed to martensite is smaller. During the friction and wear test, the contact probability between the ball and the cementite in the coating of the sample fabricated by double track is obviously higher than that of the sample fabricated by single track. Sometimes, inter-grinding of the ball and the block-like cementite occurs. This helps to enhance the wear resistance of the coating further. Therefore, the sample fabricated by double track has superior wear resistance to that of the sample fabricated by single track.

## 4. Conclusions

Based on the aforementioned research findings, PTA alloying treatment is a feasible and practical approach for surface strengthening, and the following conclusions could be drawn:

(1) Double-treatment tracks can increase the thickness of the alloy coating and the microhardness. The maximum microhardness of the sample fabricated by double track increased from 837 ± 10 HV_0.2_ for the sample fabricated by single track to 871 ± 7 HV_0.2_. Thereby extending the FeNiCrMo coating’s service life.
(2) In both samples, the coatings contain block-like and granular cementite. The block-like cementite content in the double-track PTA-treated alloy coating increases by 3.90 wt.% and the martensite content decreases by 13.04 wt.% compared to the single-track one.
(3) Both single- and double-track alloy coatings possess high hardness and wear resistance, and the sample fabricated by single track presented both abrasive and adhesive wear, and the sample fabricated by double track presented abrasive wear.

## Figures and Tables

**Table 1:** Chemical composition of rare earth fluoride (wt.%).

Rare Earth Oxide (REO)	CeO_2_	F	H_2_O
83.26	48.58	26.20	<0.50

**Table 2:** Results of semi-quantitative phase analysis.

	Single Track	Double Track
cementite	77%	80%
martensite	23%	20%

**Table 3:** Results of EDS analysis at different locations in Figure 6a,b.

Point	Element (at.%)					
	Fe	C	Si	Ni	Cr	Mo
A	63.58	30.50	3.61	1.82	0.30	0.19
B	59.33	37.74	1.21	0.65	0.82	0.24
C	59.04	36.59	2.66	1.26	0.32	0.14
D	58.09	36.64	3.20	1.81	0.22	0.04

## Data Availability

Not applicable.
